# GeoBM: A Python-based tool for integrated visualization of global bibliometric data

**DOI:** 10.1016/j.mex.2025.103497

**Published:** 2025-07-11

**Authors:** Chun Chong Fu, Jorge Fleta-Asín, Fernando Muñoz, Carlos Sáenz-Royo, Loo Keat Wei

**Affiliations:** a Department of Biological Science, Faculty of Science, Universiti Tunku Abdul Rahman, Bandar Barat, 31900 Kampar, Perak, Malaysia; b IEDIS. Departamento de Dirección y Organización de Empresas, Facultad de Economía y Empresa, Universidad de Zaragoza, Gran Vía, 2, 50005 Zaragoza, Spain; c Expert from the SIP Foundation, Zaragoza, Spain; d IEDIS. Departamento de Contabilidad y Finanzas, Facultad de Economía y Empresa, Universidad de Zaragoza, Gran Vía, 2, 50005 Zaragoza, Spain; e Departamento de Dirección y Organización de Empresas, Facultad de Ciencias Sociales y del Trabajo, Universidad de Zaragoza, Violante de Hungría, 23, 50009 Zaragoza, Spain; f Centre for Biomedical and Nutrition Research (CBNR), Universiti Tunku Abdul Rahman, Bandar Barat, 31900 Kampar, Perak, Malaysia

**Keywords:** Bibliometric analysis, Bibliometrix, VOSviewer, Citespace, Python algorithm, Country collaboration map, Production, Network, Github, Google colab

## Abstract

The rapid proliferation of scientometric and bibliometric analyses has emphasized the need for robust, scalable methods to visualize complex, large-scale research data. Conventional geospatial visualization techniques—most notably choropleth maps—often introduce significant distortions due to their inability to adequately account for spatial heterogeneity and overdispersion in bibliometric distributions. To address these methodological shortcomings, we propose GeoBM (Geographic Bibliometric Mapping), a computational framework that enables enhanced geovisualization of global scientific output and collaboration patterns. GeoBM integrates normalized country-level publication volumes with bilateral collaboration frequencies to produce high-resolution, interpretable geographic maps that reflect both research intensity and international connectivity. Implemented in Python, the framework leverages modular, algorithmically optimized routines for real-time data processing and visualization, incorporating statistical controls to mitigate overdispersion and enhance visual fidelity. The system supports extensive customization and is deployed via open-source platforms such as Google Colab and GitHub, facilitating broad accessibility and reproducibility. By providing a dual-focus representation of publication density and collaborative strength, GeoBM offers a powerful tool for the spatial analysis of global research networks, contributing to more nuanced evaluations in science policy, research management, and innovation studies.

## Highlights

• GeoBM enhances global research mapping beyond traditional choropleth limits.
• Combines publication volume and collaboration for richer geovisualization.
• Open-source, Python-based tool with real-time, customizable visual outputs.

## Graphical abstract

**Figure:**
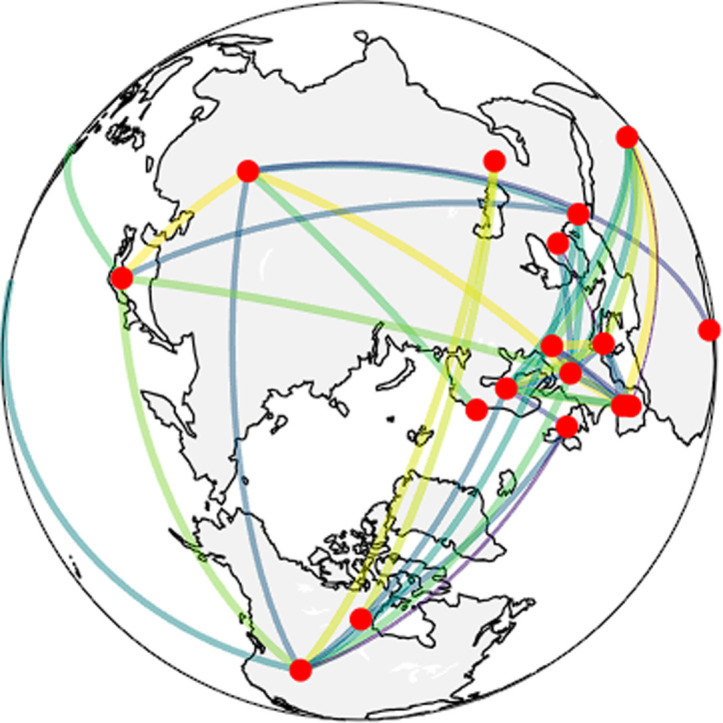

## Specifications table

**Table:** 

**Subject area**	Medical Sciences Mathematics and Statistics Economics, Econometrics and Finance Social Sciences Sciences
**More specific subject area**	Visual representation
**Name of your method**	Geographic Bibliometric Mapping [GEOBM]
**Name and reference of original method**	[1] FLEMUSA: Flexible Mapping to Understand Spatial Disparity. J. Fleta-Asín, F. Muñoz, C. Sáenz-Royo. A methodological approach and Github for enhancing visualization of country data representation in the presence of significant spatial disparity. MethodsX. 2024 (102,833) 13.
**Resource availability**	Phyton code is available in: Google colab and Github https://colab.research.google.com/drive/1NcXPybCyZu6aI8yETYahwBsFhcPi0I0k?usp=sharing *https://github.com/BGBH/Country*

## Background

Effectively visualizing spatial datasets remains a central challenge in data science, particularly when dealing with the inherent heterogeneity and overdispersion that characterize many real-world scenario. Geographic data in fields such as bibliometrics and scientometrics often exhibit highly skewed patterns, with a small number of regions contributing disproportionately to global research output and collaboration networks. Traditional visualization methods, most notably choropleth maps, frequently fail to capture these disparities accurately. Such methods are susceptible to visual distortion, leading to misleading representations that obscure critical variations and trends. These limitations emphasize the urgent need for advanced geospatial visualization techniques that can accommodate the complex statistical properties of bibliometric data while preserving geographic integrity and interpretability [1].

In response to these challenges, this article introduces a novel Python-based algorithm that provides a flexible, accessible, and high-fidelity framework for visualizing spatial bibliometric data. Grounded in classical cartographic principles that date back to Ptolemaic geography, the approach adapts and extends the methodology proposed by Fleta-Asín et al. [1], originally developed to address spatial distortion in economic data visualization. Our framework recontextualizes their method within the domain of bibliometrics, enhancing it to accommodate the multidimensional structure of publication and collaboration data. By leveraging widely available open-source platforms such as Google Colab, the tool supports real-time interactivity, user customization, and collaborative workflows, democratizing access to sophisticated spatial analytics for a broad research audience.

The growing prominence of bibliometric and scientometric studies has intensified the demand for visualization tools that can synthesize large-scale, multifaceted datasets into interpretable visual representations [[2], [3], [4], [5]]. Despite the proliferation of existing platforms, several key limitations persist. For instance, Bibliometrix R/Biblioshiny [6] allows for the generation of country-level production and collaboration maps but lacks integrated indicators for publication frequency and often produces cluttered visuals as data complexity increases. Moreover, its collaboration maps employ simple connecting lines that fail to convey interaction strength, necessitating additional data interpretation through external tables. VOSviewer [7], although proficient in quantifying collaboration strength via network diagrams, omits geographical context, making it unsuitable for spatial analysis. CiteSpace [8], while powerful in detecting citation bursts and mapping intellectual structures, lacks built-in mechanisms for country-level or geographically explicit analysis, limiting its applicability to global-scale assessments.

To address these gaps, we present a unified methodology that enables simultaneous representation of publication volume, frequency, and collaborative intensity within a single, coherent geographic visualization. The proposed algorithm integrates spatial topology with quantitative bibliometric indicators, allowing users to identify regional disparities, collaborative hubs, and emerging research centers with improved clarity. Emphasis is placed on modularity and scalability, allowing the framework to be adapted for various dataset sizes and research objectives. Through this integration of spatial analytics and bibliometric data, our approach advances the methodological toolkit available to researchers, enabling richer interpretations of global scientific activity. By overcoming the technical constraints of existing tools and fostering open, reproducible workflows through Python and cloud-based platforms, our solution represents a significant step forward in the visualization and interpretation of bibliometric landscapes.

## Method details

This study employs an integrative geospatial bibliometric approach to visualize and analyze global patterns of scientific productivity and international collaboration. To facilitate this, we developed GeoBM—an open-source, Python-based analytical framework capable of processing bibliometric data and rendering spatially explicit network visualizations. GeoBM is designed to accommodate datasets exported from major bibliographic databases such as Web of Science (WOSCC) [9], Scopus, and Dimensions, and is compatible with data formats generated via the Bibliometrix R package. For the purpose of demonstrating the methodological workflow, a synthetic dataset was constructed to replicate real-world bibliometric structures, encompassing publication volumes and inter-country co-authorship relationships among 195 sovereign states.

The analytical workflow comprises four interdependent stages: (1) data preprocessing and harmonization, (2) spatial representation of publication volumes, (3) modeling of bilateral scientific collaboration, and (4) integrative network visualization in both planar and three-dimensional projections. The pipeline leverages a suite of open-source Python libraries, including pandas for data manipulation, geopandas and cartopy for geospatial processing, matplotlib for static visualizations, and optionally plotly for interactive 3D rendering. The modularity of the codebase ensures scalability and reproducibility across a wide range of bibliometric datasets.

In the first stage, bibliometric data are parsed and normalized to derive country-level publication counts, which are assigned based on the affiliations of corresponding authors—a widely accepted proxy for national scientific output. These publication metrics are then geocoded and spatially joined to a standardized set of geopolitical boundaries using ISO 3166–1 alpha-3 country codes. Geospatial boundary data are sourced from publicly available shapefiles, which are reprojected and generalized as necessary to ensure cartographic clarity. The harmonized dataset is prepared in a geodataframe structure, enabling seamless integration of bibliometric and spatial attributes.

**Fig. 1:**
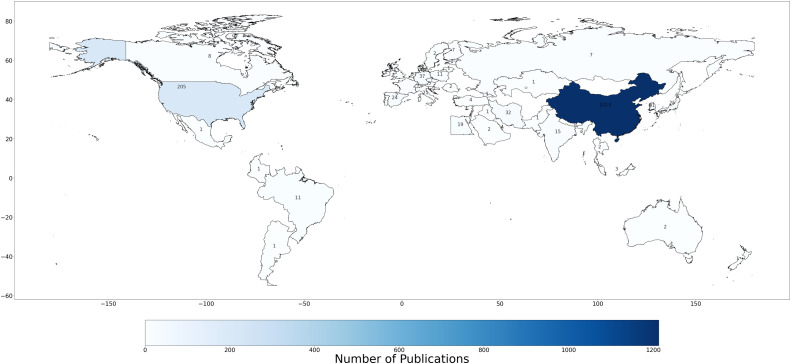
Publication volumes ranked based on corresponding author’s country.

The second stage involves the construction of a global choropleth map to visualize national publication output (Fig. 1). Countries are shaded using a monochromatic blue gradient, wherein darker hues denote higher levels of scientific productivity. Numeric labels are overlaid on each country to indicate absolute publication counts, thereby facilitating both comparative and absolute interpretation. This representation reveals spatial disparities in global knowledge production, with dominant contributions observed from China, the United States, and selected European nations.

**Fig. 2:**
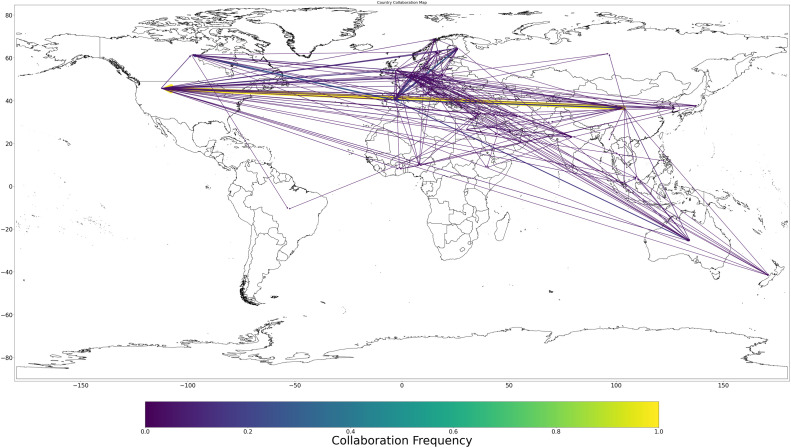
Collaboration frequencies among countries.

The third stage of the analysis models international scientific collaboration through a co-authorship network. In this undirected, weighted network, nodes represent countries, while edges correspond to the frequency of co-authored publications between each pair of nations. Collaboration strength is encoded using a normalized metric ranging from 0 to 1, and visualized through edge thickness and a color gradient based on the perceptually uniform viridis colormap (Fig. 2). This stage uncovers the structural topology of the global collaboration landscape, revealing densely connected research blocs and identifying central versus peripheral actors in international science.

**Fig. 3:**
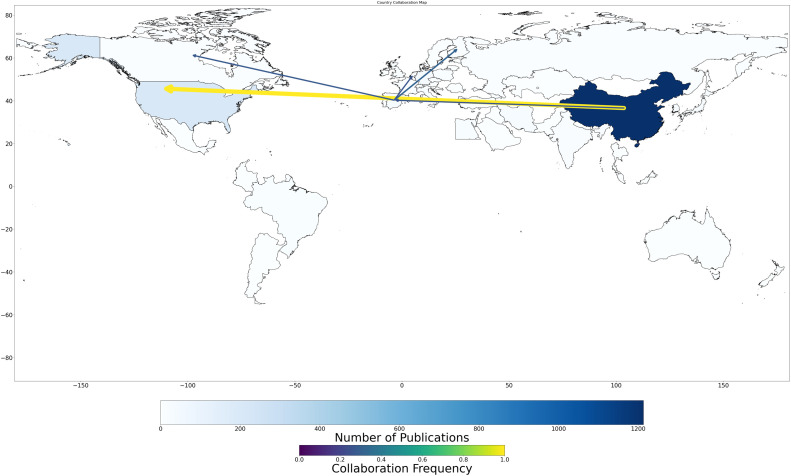
Publication volumes and collaboration frequencies among countries.

In the fourth stage, publication volume and collaboration intensity are jointly visualized in an overlaid network-choropleth composite (Fig. 3). This integrative map locates the collaboration network atop the publication output base layer, allowing for multidimensional interpretation. The fusion of these two dimensions enables users to distinguish between countries that are highly productive but insular, and those that are less prolific yet deeply embedded in global research networks. For instance, the juxtaposition highlights instances of scientific "bridge nations" that, despite modest output, it serves as crucial connectors within the global knowledge ecosystem.

**Fig. 4:**
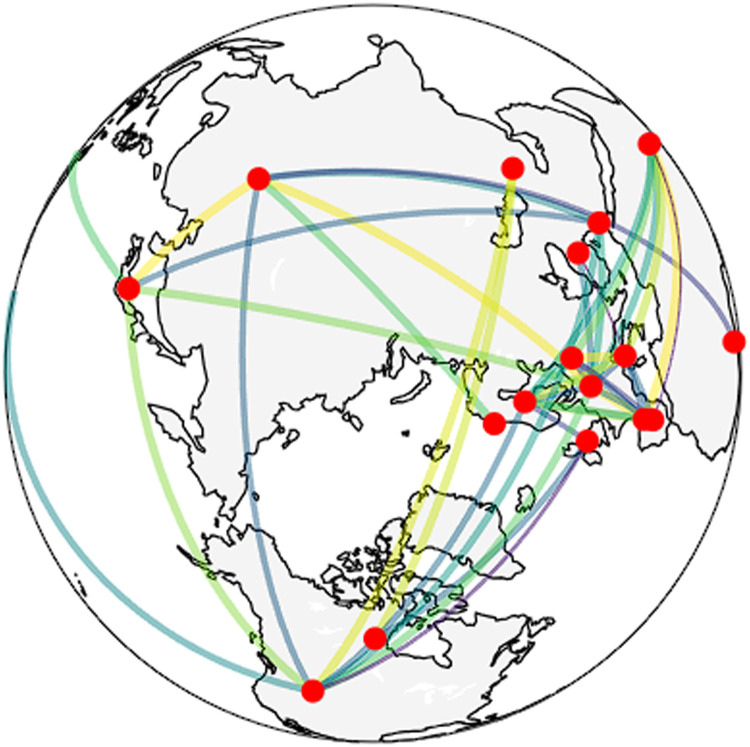
Three-dimensional geospatial representation of global scientific collaboration networks.

To address the limitations of planar cartographic projections—particularly with respect to long-distance or trans-hemispheric collaboration—a final visualization (Fig. 4) re-projects the collaboration network onto a three-dimensional globe. Using great-circle geometry, collaboration links are rendered as geodesic arcs that follow the Earth's curvature, offering a topologically faithful depiction of global connectivity. Red nodes represent collaborating countries, while arc colors encode normalized collaboration intensity. This immersive representation enhances the spatial interpretability of global networks and minimizes distortion inherent in two-dimensional projections, particularly for collaborations spanning the Pacific and Arctic regions.

The entire pipeline is fully reproducible and accessible via GitHub, with an executable version available on Google Colab to facilitate immediate deployment without local dependencies. By integrating bibliometric analysis with geospatial science, GeoBM offers a novel methodological contribution that bridges scientometrics, cartography, and data visualization. This approach is particularly valuable for policy-makers, research administrators, and scholars seeking to examine the spatial dimensions of knowledge production and trans-national collaboration in the evolving global research landscape.

## Methodological innovation and comparative advantage of GeoBM in bibliometric visualization

GeoBM represents a methodological breakthrough in bibliometric visualization by addressing persistent limitations in existing mapping and analysis tools. Traditional platforms often isolate research productivity from collaborative patterns, leading to fragmented or one-dimensional representations that obscure the nuanced structure of global scientific activity. In response, GeoBM introduces an integrated geospatial framework that merges these dimensions, allowing for a simultaneous, spatially grounded visualization of both publication output and international collaboration. It achieves this through a dual-layered design in which country-level research productivity is encoded using choropleth-style shading, while bilateral collaborations are depicted as dynamically scaled, directed arrows. These arrows vary in thickness and color gradient according to normalized collaboration frequencies, producing a cohesive, data-rich visualization of global research interconnectivity (see Fig. 2, Fig. 3).

The framework is technically optimized for both performance and accessibility. Developed entirely in Python and leveraging open-source libraries such as pandas, geopandas, numpy, matplotlib, and plotly, GeoBM is platform-independent and deployable in cloud environments like Google Colab. Its lightweight computational architecture enables the production of publication-quality maps in under a minute—often in as little as 15 seconds—making it highly suitable for iterative analysis and rapid data exploration. Importantly, GeoBM is open-source and freely available on GitHub, requiring no proprietary software or local installation. Input data can be seamlessly prepared using existing tools like Bibliometrix, allowing for users with minimal programming experience to conduct advanced geospatial analysis through simple Excel-based files.

One of GeoBM’s defining strengths is its modular and customizable design, which allows researchers to tailor visualizations to diverse analytical objectives. Visual parameters—such as color schemes, node sizes, arrow scaling, and spatial filters—can be easily adjusted to highlight regional disparities, spotlight emerging scientific hubs, or analyze asymmetrical collaboration flows. This flexibility is particularly advantageous for comparative research, including cross-country evaluations, longitudinal studies, and assessments of geographic equity in science. Crucially, GeoBM avoids the graphical overload common in dense network visualizations by employing dynamic scaling and directional encoding. These features preserve interpretability even when rendering highly complex, data-dense global networks.

Beyond its visual sophistication, GeoBM provides critical analytical utility in an era of exponentially expanding scientific output and increasingly globalized research collaboration. As bibliometric datasets grow in volume and granularity, the need for tools that can preserve spatial fidelity becomes ever more urgent. GeoBM meets this need by aligning with the FAIR (Findable, Accessible, Interoperable, and Reusable) data principles, offering a transparent and scalable methodology [10]. Its capacity to reveal scientific hotspots, highlight collaboration asymmetries, and surface underrepresented regions makes it particularly valuable for policy development, institutional benchmarking, and strategic planning in global science. Moreover, GeoBM serves as a robust foundation for future innovation in bibliometric analysis. Its architecture supports extensible capabilities such as interactive dashboards, real-time data integration, machine learning-driven trend detection, and temporal animations of collaboration networks. These enhancements would significantly expand its utility in dynamic research environments, from live monitoring of research ecosystems to foresight analysis for science and innovation policy.

When compared to leading bibliometric platforms such as CiteSpace, VOSviewer, and Bibliometrix/Biblioshiny, GeoBM offers clear methodological and functional advantages, particularly in the geospatial domain. CiteSpace excels in detecting citation bursts and conceptual clusters and in visualizing the temporal evolution of scientific fields. However, its focus on intellectual structure comes at the expense of geographic context; it lacks native support for spatial mapping and thus cannot effectively represent global disparities or cross-national research dynamics [7]. Similarly, VOSviewer is widely praised for its high-quality visualizations of co-authorship, citation, and keyword networks. Yet, its reliance on force-directed layouts abstracts away geographic origin, preventing users from interpreting collaboration patterns within a real-world spatial framework. While the tool provides quantitative indicators of collaboration strength, these are not geographically contextualized, thereby limiting its relevance for regional policy analysis or international cooperation strategy [8]. Bibliometrix and its graphical interface Biblioshiny offer some geospatial capabilities, such as world maps of publication density and collaboration matrices [6]. However, these tools often fall short in analytical depth and graphical clarity. Their visualizations lack proportional scaling for collaboration intensity, suffer from visual clutter when applied to dense networks, and are constrained by limited customization options within the R Shiny environment. Integrating more sophisticated spatial operations typically requires advanced programming expertise and extensive manual processing [[6], [7], [8]].

In contrast, GeoBM seamlessly integrates geographic precision with analytical rigor. It grounds bibliometric entities in real-world coordinates using shapefiles, thereby enabling accurate spatial interpretation of both scientific production and international collaboration. Its dual-dimensional visualization approach—combining choropleth shading for publication volume with scaled, directional arrows for collaboration—offers a holistic view of scientific activity that is both interpretable and richly informative. The system is also highly scalable, capable of rendering complex, high-density datasets without sacrificing clarity, thanks to intelligent visual filtering and responsive design. Unlike the comparatively rigid architectures of other platforms, GeoBM is fully open-source, cloud-compatible, and highly configurable, allowing researchers to adapt it to varied data sources, workflows, and presentation needs. Furthermore, GeoBM is built for performance and reproducibility. It supports rapid rendering and deployment in collaborative, cloud-based environments, making it ideal for time-sensitive analyses and multi-institutional projects. Its extensibility enables seamless integration with emerging technologies, from machine learning pipelines to interactive data visualizations, positioning it as a forward-looking solution for scientometric research.

In summary, GeoBM represents a significant advancement in the field of bibliometric mapping and analysis. By fusing geographic accuracy with analytical depth, and combining technical efficiency with user-centered design, it redefines how global scientific activity can be visualized, interpreted, and acted upon. It not only empowers researchers and institutions with high-resolution, interpretable, and reproducible visualizations, but also sets a new benchmark for open, extensible, and equitable bibliometric tools. As the architecture of global science continues to evolve, GeoBM stands as a vital instrument for capturing and understanding the spatial dynamics that shape the future of knowledge production.

## Limitations and future studies

While the current algorithm effectively enhances bibliometric visualization, it faces limitations such as reliance on data accuracy, potential visual clutter in dense networks, limited accessibility for non-coders, simplified geographic representations, and a narrow scope of bibliometric analyses.

Future studies should prioritize the development of automated data-cleaning and validation tools to improve input quality. Addressing visual clutter can be achieved by incorporating advanced interactive features like multi-scale zooming, attribute-based filtering, and dynamic layer toggling to enable more precise network exploration. Enhancing usability through the creation of a user-friendly Graphical User Interface (GUI) or standalone application will broaden adoption among researchers without programming expertise. Furthermore, refining spatial analysis by integrating region-specific geospatial projections will improve geographic accuracy. Finally, expanding the algorithm to include advanced bibliometric methods such as citation burst detection, temporal trend analysis, and clustering algorithms will substantially increase its analytical depth and versatility. These research directions will collectively advance the algorithm’s precision, accessibility, and overall utility in bibliometric studies.

## Ethics statements

Our study did not involve human subjects, animal experiments, or data collected from social media platforms. The work presented is based on the development and application of computational algorithms for bibliometric visualization using publicly available datasets. All data used were obtained from openly accessible sources, and no personally identifiable or sensitive information was involved. The methodologies applied comply with ethical research standards and adhere to the principles of transparency, reproducibility, and responsible data handling.

## CRediT author statement

Chun Chong Fu: Methodology, Software, Validation, Formal analysis, Visualization. Jorge Fleta-Asín: Software, Writing - review & editing, Validation, Visualization, Funding acquisition. Fernando Muñoz: Software, Writing - review & editing, Validation, Visualization, Funding acquisition. Carlos Sáenz-Royo: Software, Writing - review & editing, Validation, Visualization, Funding acquisition. Loo Keat Wei: Data curation, Writing – Original Draft, Writing - review & editing, Methodology, Validation, Visualization, Supervision, Project administration, Funding acquisition. All authors revised the drafts and approved the final proof.

## Declaration of competing interest

The authors declare that they have no known competing financial interests or personal relationships that could have appeared to influence the work reported in this paper.

## Data Availability

Data will be made available on request.
